# High-level heterologous production and Functional Secretion by recombinant *Pichia pastoris* of the shortest proline-rich antibacterial honeybee peptide Apidaecin

**DOI:** 10.1038/s41598-017-15149-3

**Published:** 2017-11-06

**Authors:** Ximing Chen, Juan Li, Haili Sun, Shiweng Li, Tuo Chen, Guangxiu Liu, Paul Dyson

**Affiliations:** 1 Key Laboratory of Desert and Desertification, Northwest Institute of Eco-Environment and Resources, Chinese Academy of Sciences, Lanzhou, China; 2 Key Laboratory of Extreme Environmental Microbial Resources and Engineering of Gansu Province, Jiayuguan, China; 3 School of Chemistry and Environment Science, Lanzhou City University, Lanzhou, China; 4 School of Chemical and Biological Engineering, Lanzhou Jiaotong University, Lanzhou, China; 5 Institute of Life Science, Swansea University Medical School, Singleton Park, Swansea, SA2 8PP UK

## Abstract

Drug resistance is a major problem in antibacterial chemotherapy. Apidaecins, which refer to a series of small, proline-rich antimicrobial peptides, are predominantly active against many drug-resistant bacteria. The apidaecins have special antibacterial mechanisms, and are non-toxic for human cells, a prerequisite for using them as novel antibiotic drugs. However, no efficient non-tagged apidaecin expression system has been reported, which is the limiting factor for their application. Here we successfully generated a *Pichia pastoris* transformant expressing and secreting apidaecin. However, expression was unstable and poor. Analysis of this revealed that the integration plasmid was frequently lost and that apidaecin expression resulted in cell death. Using N-methyl-N-nitro-N-nitroso-guanidine mutagenesis and selection, a mutant strain Apmu4 was derived, in which the rate of loss of the integration plasmid was much lower after induction, and which produced improved titres of apidaecin. Additionally, we discovered that using glucose as the sole carbon source to pre-culture the strain before induction could greatly enhance apidaecin production. A pilot-scale 10 L fermentation yielded 418 mg/L of recombinant apidaecin, which represents the highest reported yield of apidaecin. Consequently, this study reports the first super heterologous expression and secretion of apidaecin in yeast.

## Introduction

Antimicrobial peptides are evolutionarily ancient weapons, which appear to be ubiquitous and multipotent components of the innate immune defense arsenal used by both prokaryotic and eukaryotic organisms^1,2^. Despite great differences in size, amino acid composition and structure, most of the antimicrobial peptides from insects can be grouped into three categories. The largest category in number contains peptides with intramolecular disulfide bonds forming hairpin-like **β**-sheets or **α**-helical-**β**-sheet mixed structures. The second most important group is composed of peptides forming amphipathic **α**-helices. The third group comprises peptides with an overrepresentation in proline and/or glycine residues^3^. These proline-rich antibacterial peptides can inhibit an intracellular target in bacteria without destroying or remaining attached to the bacterial cell membrane, and as such have emerged as viable candidates for the treatment of mammalian infections, and so are of particular interest as potential new antimicrobial drugs^4^.

The apidaecins are a series of small, proline-rich (Pro-rich), 18 to 20 residue peptides produced by insects^5^. Three isoforms of apidaecins, HbIa, HbIb and HbII, were first isolated from lymph fluid of honeybees (*Apis mellifera*) that were infected with bacteria^6^. Unlike most conventional antibiotic peptides that are amphipathic, apidaecins are non-amphipathic and may have better membrane penetration ability. The most unique feature about their mode of action is a total lack of pore-forming activity^5^. The honeybee-derived apidaecins are lethal to many Gram-negative bacteria such as *E. coli*, *Enterobacter cloacae*, *Klebsiella pneumonia*, *Salmonella typhimurium*, *Salmonella typhi*, *Shigella dysenteriae*, *Acinetobacter calcoaceticus* and *Agrobacterium tumefaciens* ^7^. Apidaecins have been recognized as potential therapeutic alternatives to antibiotics because of their immediate effect, their apparent nontoxicity toward eukaryotic cells, and the fact that there is little or no bacterial resistance^8,9^.

In terms of their medical use, the extraction of apidaecins from bees is very expensive. Only 100 mg of native apidaecins can be routinely purified from a batch of 5000 bees^6^. Additionally, chemical synthesis of apidaecins is also economically disadvantageous, with production costs of 100 mg (≥80% purity) of chemosynthetic apidaecins being at least 580 USD. Recombinant apidaecins, fused with *Streptomyces* subtilisin inhibitor, ubiquitin or nisin, have been successfully expressed in *Streptomyces* sp., *E. coli* and *Lactococcus lactis*, respectively^10–13^. However, low production yields and complex purification procedures of these recombinant apidaecins are limiting factors for these systems. As expression systems based on *Pichia pastoris* have been used successfully for the production of various recombinant heterologous proteins^14^ including some bacteriocin peptides EntL50A and EntL50B^15,16^ and plectasin-derived peptides^17,18^, this offers a potential improved system for synthesis of recombinant apidaecin. This expression system has many advantages such as ease of genetic manipulation, inexpensive culture to high cell densities, posttranslational modifications of proteins, no toxicity from intracellularly accumulated material, and easy purification with very low secretion of endogenous proteins^19–21^.

Here we successfully generated *P. pastoris* transformants which can be induced by methanol to express and secrete apidaecin. We determined that there are several factors that impact on apidaecin yield using this system. One is the loss of the integration plasmid and another is cell death following induction of expression of apidaecin. After N-methyl-N-nitro-N-nitroso-guanidine (NTG) mutagenesis and selection, a significantly higher apidaecin production mutant strain APmu4 was derived. The rates of plasmid loss and cell death were both much lower in this high yielding strain compared to the progenitor. In addition, we determined that protein degradation is another limiting factor for apidaecin production. Inclusion of peptone and yeast extract in the growth medium can effectively protect against proteolysis of apidaecin following induction.

## Results

### Biologically active apidaecin produced by *P. pastoris*

**Figure 1:**
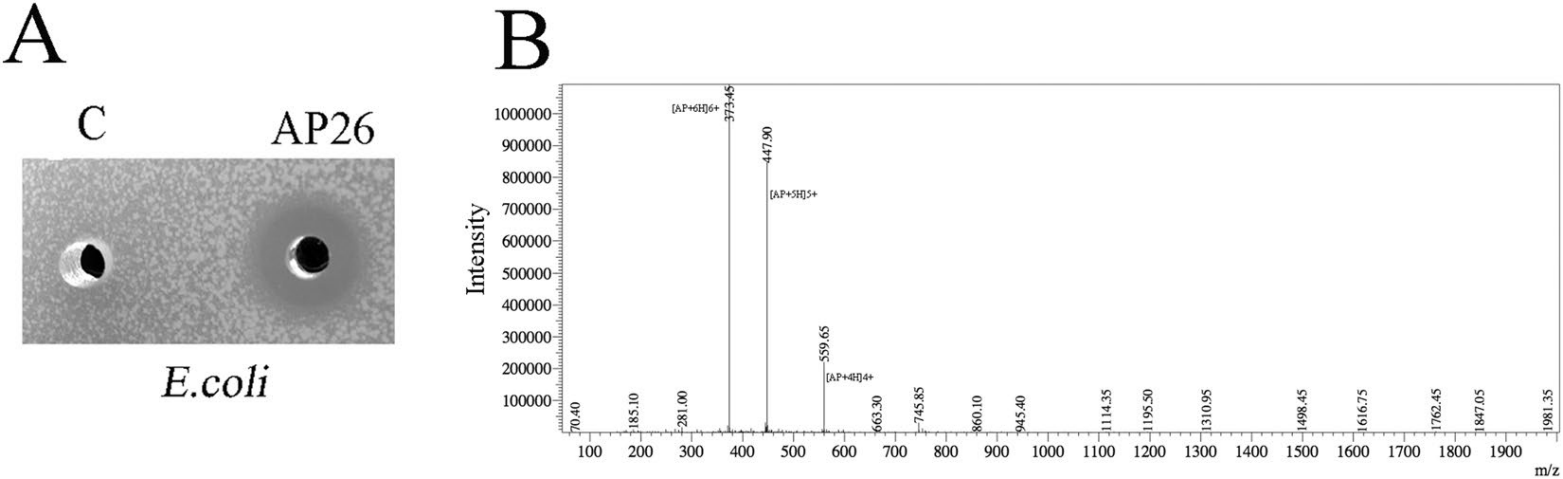
Construction of recombinant apidaecin produced by *P. pastoris*. (**A**) Antimicrobial activity of *P. pastoris* AP26 (labeled as AP26) and *P. pastoris* C (labeled as C) fermentation supernatants, sampled 72 h after induction, against *E.coli*. (**B**) ESI-MS analysis of the apidaecin fermentation supernatants.

Previous studies demonstrated that N-terminal mutant forms of apidaecin have stronger activity to inhibit gram-negative bacteria compared to the wild type apidaecin^22^. Of these mutant forms of apidaecin, the peptide 1C-20, with the amino acid sequence RVRRPVYIPQPRPPHPRL, has the strongest anti-bacterial ability. Consequently, we chose this this peptide for heterologous expression in *P. pastoris*. A yeast clone, *P. pastoris* AP26, was constructed to produce this apidaecin, in addition to the antimicrobial peptide (AMP)-negative control clone *P. pastoris* C. In the clone, MF**α**1 s and the Kex2 sequence were fused in frame to the apidaecin gene to guide the proper processing and secretion of the mature apidaecin peptide. 72 h after induction with methanol, the culture supernatant from AP26 displayed antimicrobial activity to inhibit *E.coli* JM109, a strain which exhibits some resistance to apidaecins. However, no zone could be detected with the supernatant from the negative control strain, *P. pastoris* C (Fig. 1A). After ESI-MS of the fermentation supernatant of AP26, fragmentation peaks specific to apidaecin were detected, giving a combined molecular weight of 2234.7 Da, which conformed to the theoretical value of 2233.75 (Fig. 1B).

### The loss of the integration plasmid and cell viability are limiting factors that affect yields of recombinant apidaecin

**Figure 2:**
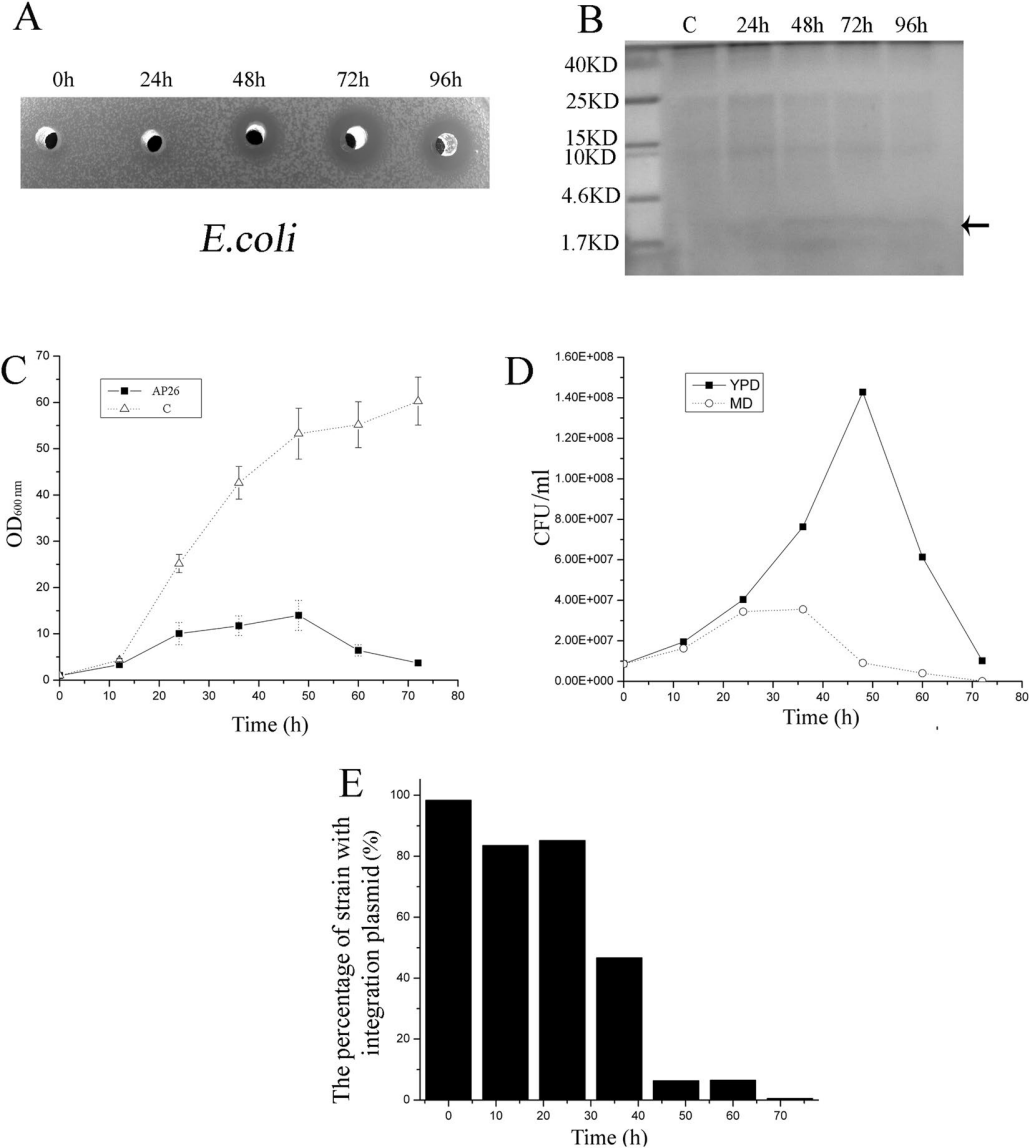
The loss of the integration plasmid is a key factor affecting apidaecin expression. (**A**) Antimicrobial activity of fermentation supernatants (50-μl) sampled at different time points after induction (0, 24, 48, 72 and 96 h) against *E.coli*. (**B**) Tricine-SDS–PAGE analysis of apidaecin in fermentation supernatants. The left-hand lane was loaded with 10 μl of a protein molecular weight marker. Lane C: 10 μl of *P. pastoris* C strain 72 h fermentation supernatants. Lane 24 h–96 h: 10 ul of AP26 fermentation supernatants taken at 24 h, 48 h, 72 h and 96 h. (**C**) Strains AP26 and C were first grown in MD liquid medium at 30 °C for 48 h and then transferred into BMMY buffered methanol complex medium, with both cell concentrations at OD600 = 1 at the beginning. The growth curves were measured at 0, 24, 48 and 72 h respectively. (**D**) Following methanol induction, the CFU/ml of AP26 grown on MD and YPD medium were measured at 0, 24, 48 and 72 h respectively. (**E**) The percentage of integration plasmid loss of AP26 was calculated following methanol induction.

Previous studies showed the production of some AMPs (such as EntL50A, EntL50B and EntL50C) is not stable following methanol induction in *P. pastoris* ^16^. We observed that the antimicrobial activity of apidaecin in culture supernatants of *P. pastoris* AP26 could be detected 48 h after methanol induction and the maximum antimicrobial activity was detected 72 h after methanol induction. However, the antimicrobial activity then decreased 96 h after induction (Fig. 2A). These activities were correlated with weak bands of apidaecin that could be detected after Tricine-SDS–PAGE at 48 h, 72 h and 96 h (Fig. 2B). To examine the apparent decline of apidaecin production, we measured cell viability and retention of the integration plasmid. Figure 2C indicates that following induction the cell concentration of *P. pastoris* AP26 increased slowly, reaching a maximum at 48 h. After this the cell concentration gradually decreased. In comparison, the cell concentration of control strain C always increased rapidly. This decline in cell viability was mirrored in cell counts after plating on YDP medium (Fig. 2D). Viable cells that maintain the integration plasmid can grow and form colonies on MD medium, whereas cells which lose the plasmid cannot grow on this medium. Cell counts on MD medium following methanol induction revealed significant loss of the integration plasmid (Fig. 2D). Indeed, after 48 h induction most of the viable cells contained no integration plasmid (Fig. 2E). In comparison, we observed no plasmid loss in the control strain C following induction (data not shown). Previous studies have indicated that yeast can lose the integration plasmid when it encodes a toxic compound^23^. Therefore we interpreted our data to imply that expression of apidaecin is toxic to *P. pastoris* and this induces the loss of the integration plasmid.

### Selecting a higher apidaecin-production mutant with lower integration plasmid loss

**Figure 3:**
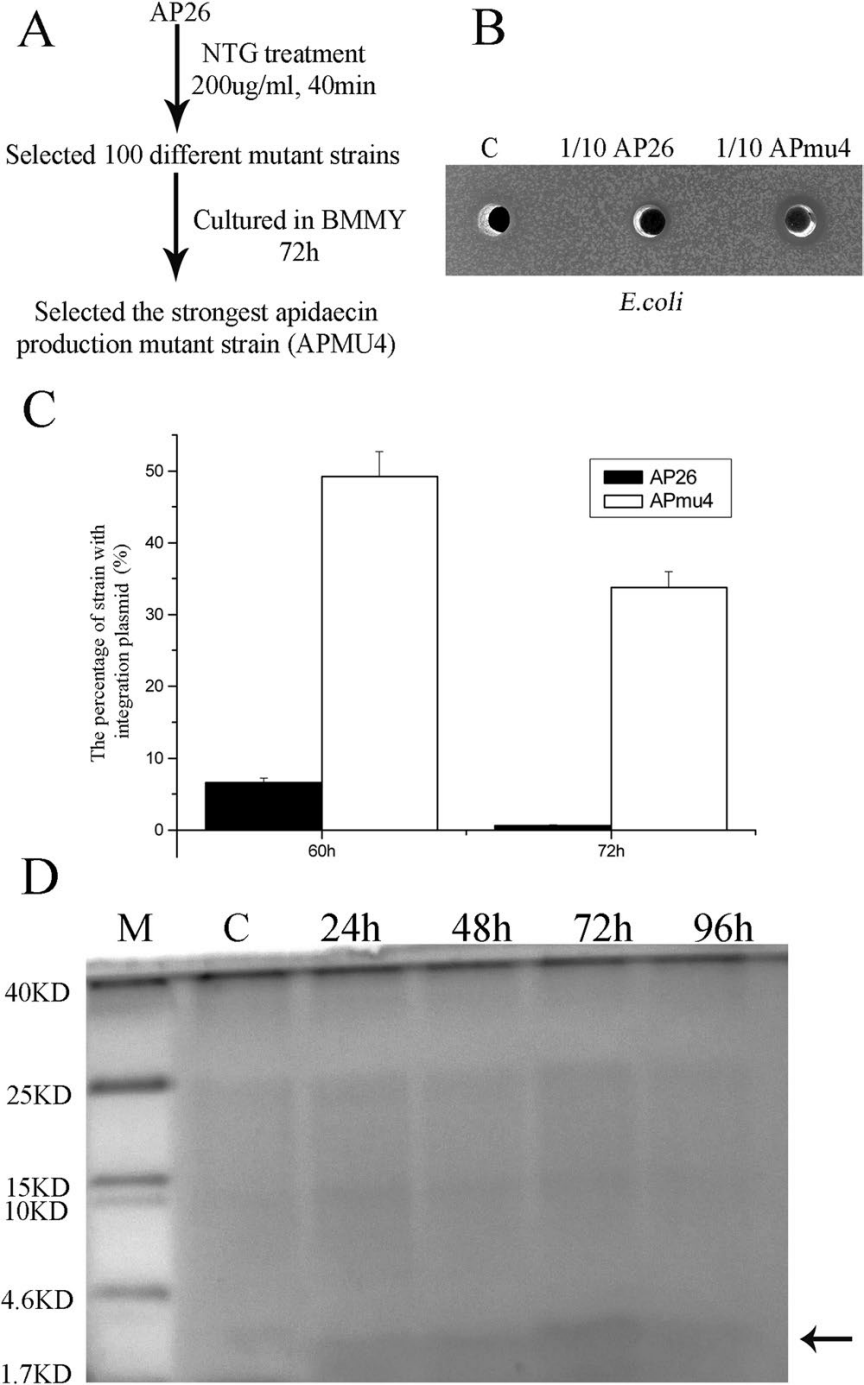
Selecting a higher apidaecin-production mutant with lower integration plasmid loss. (**A**) NTG mutagenesis was used to select a higher apidaecin-production mutant strain. (**B**) Antimicrobial activity of AP26, C and APmu4 fermentation supernatants, sampled 72 h after induction and diluted 10-fold in the water, against *E.coli*. (**C**) The percentage of integration plasmid loss rates of AP26 and APmu4 were calculated at 60 and 72 h respectively. (**D**) Tricine-SDS–PAGE analysis of apidaecin in fermentation supernatants. Lane M: 10 μl of protein molecular weight marker. Lane C: 10 μl of *P. pastoris* C strain 72 h fermentation supernatants. Lane 24h-96h: 10ul of APmu4 fermentation supernatants taken at 24 h, 48 h, 72 h and 96 h.

Previous studies have indicated that gene mutations can confer resistance to apidaecin in bacteria^24^. We employed NTG mutagenesis, exposing *P. pastoris* AP26 for 40 min, and then cultured the mutant library on YPD medium (Fig. 3A). 100 different mutant clones were then cultured on BMMY medium for 72 h, and the strongest apidaecin production mutant strain (*P. pastoris* APmu4) was selected (Fig. 3B). To address the basis for the higher production of apidaecin, we analysed the rate of plasmid loss, indicating that, following induction of apidaecin synthesis, the plasmid is much more stable in *P. pastoris* APmu4 compared to AP26 (Fig. 3C). Analysis of culture supernatants by Tricine-SDS–PAGE revealed a higher abundance of apidaecin produced by the mutant, reaching a maximum after 72 h fermentation following induction (Fig. 3D). To address whether stability of apidaecin due to proteolysis was affected in the mutant, 100 ug of chemical synthetic Ap was combined with 1 ml of supernatants from cultures of AP26 and APmu4 sampled 96 h after methanol induction. The reactions were incubated at 30 °C for 24 h. Table S3 indicates rapid loss of antimicrobial activity due to proteolytic activities derived from both strains. These data imply that the basis for higher yields of apidaecin by APmu4 is due to higher expression due to higher retention of the integration plasmid encoding the gene.

### Optimization of apidaecin yields

The optimal culture conditions for apidaecin expression were determined using shake flasks. Previous studies showed the glucose inhibits the activity of the AOX1 promoter^25^. Here we compared the effect of different carbon source before the induction on the apidaecin expression. Before methanol induction, independent cultures of Apmu4 were grown in BMGY and BMGluY. After growth, the strains were harvested by centrifugation and resuspended in BMMY medium. Following 72 h induction, the pre-culture grown in the BMGluY medium produced the highest yield of apidaecin (Table S4). These data suggest pre-culturing in medium with glucose, which decreases the background expression of apidaecin, is good a strategy for growing up the strain prior to induction. Using this method to culture strain AP26 still resulted in much lower yields of apidaecin compared to the high-producing mutant Apmu4 (Table S5).

### Pilot-scale fermentation of apidaecin

The typical fermentation process was composed of three steps: a batch phase, carbon feeding phase and methanol induction phase. During the batch phase, the strain was cultured in the YDFM medium contained 3% glucose (pH 5.0) and controlled at 28 °C. The batch phase usually lasted 19–22 h and ended when the dissolved oxygen (DO) increased rapidly, which indicated depletion of glucose. At this time, the cell wet weight reached approximately 92 g/L. During the glucose feeding phase, 50% glucose supplemented with 12 ml/L PTM1 solution was supplied through feeding, and DO was controlled at 30% by limiting airflow. The total glucose concentration for the feeding was 50 g/L added to the medium. An this time, the cell wet weight reached approximately 156 g/L. At the end of the glucose feeding phase, the strain was cultured for 2 h with no carbon source feeding, to deplete the remaining glucose in the medium. Before methanol induction, the pH value was adjusted to 6.8, which is the optimum pH for the protein expression by *Pichia pastoris* ^26^ and tryptone and yeast extract were added to final concentrations of 20 g/L and 10 g/L, respectively. The methanol induction phase was started by a stepwise increase in the methanol-feeding rate (100% methanol with 12 ml/L PTM1 salts) from 3.6 ml/hr/liter to 10.9 ml/hr/liter over 5–8 h. The DO was restricted to approximately 30% by limiting the supply of methanol and oxygen. The apidaecin activity was detected 12 h after induction (Table S6). At this time, the cell wet weight reached 195 g/L. 72 h after induction, the apidaecin activity reached 5,740,361 AU/ml.

### Purification and characterization of apidaecin

**Figure 4:**
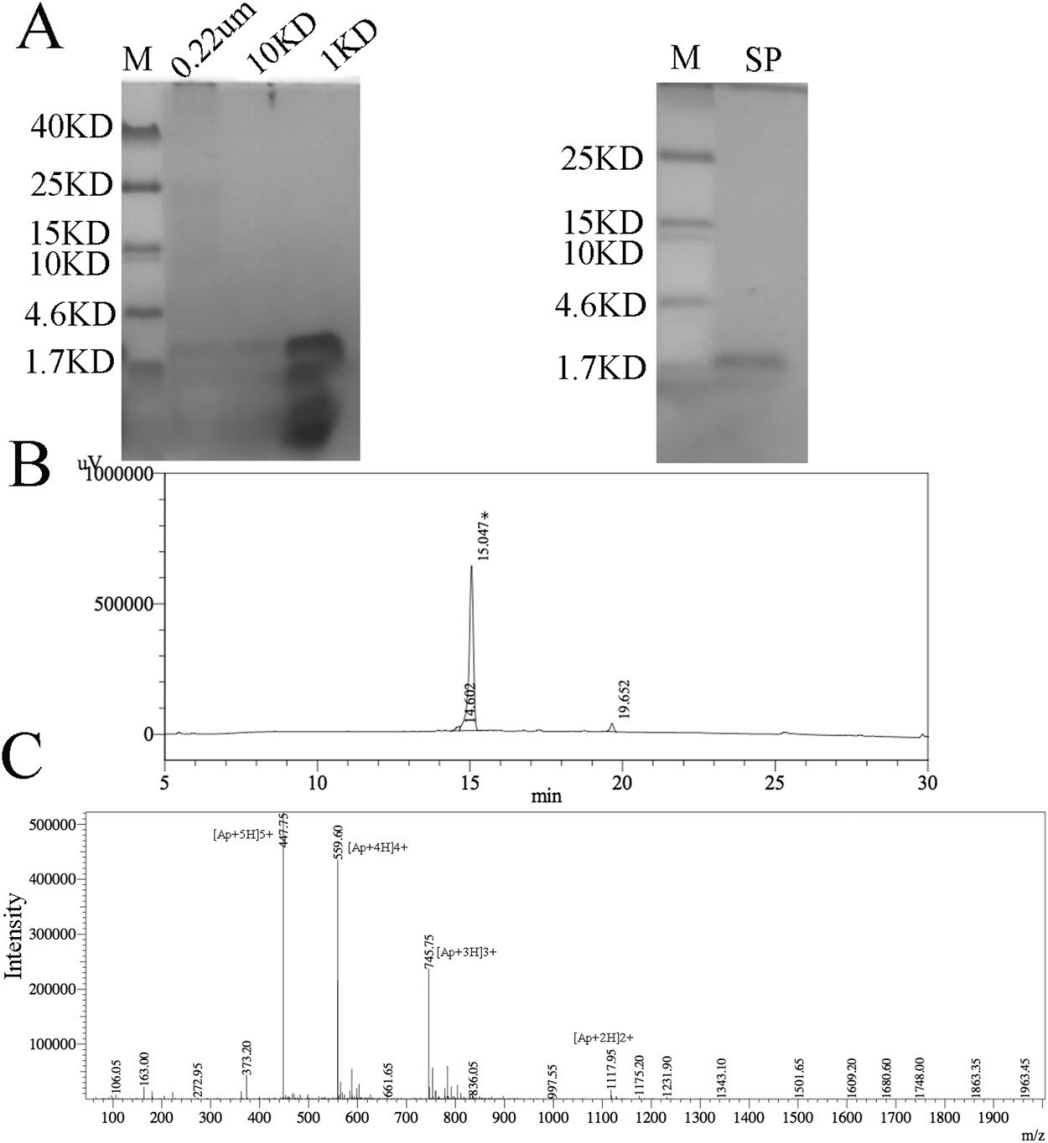
Purification and identification of apidaecin. (**A**) The samples from each step during the purification processes were monitored by Tricine-SDS–PAGE. (**B**) Protein fractions with antimicrobial activity against *E. coli* eluted from an SP FF column were further purified by HPLC at a flow speed of 1 ml/min. Buffer A for the HPLC was 0.1% trifluoroacetic in 100% water. Buffer B for the HPLC was 0.1% trifluoroacetic in 100% acetonitrile. (**C**) Apidaecin was detected by ESI-MS from the HPLC fraction denoted with an asterisk.

Protein from 1 L of fermentation supernatant was purified by two steps: filtration and capturing apidaecin by ion-exchange chromatography. First the supernatant was filtrated using 0.22 um hollow-fiber membranes. Second the proteins were concentrated using 10 kDa and 1 kDa hollow-fiber membranes and diluted 1:4 with low salt buffer (20 mM sodium phosphate buffer, pH 7.5) and purified by anion exchange chromatography with fractions eluted with buffer B (0.4 M NaCl, 20 mM sodium phosphate buffer, pH 8.0). The fractions from each step were monitored for antibacterial activity (Table S7). Tricine-SDS–PAGE indicated co-purification of other proteins together with apidaecin after concentration with 10 kDa and 1 kDa hollow-fiber membranes. After anion exchange chromatography, only one band could be detected after Tricine-SDS–PAGE (Fig. 4A). Using this process, the final products surpassed 95% purity as determined by HPLC (Fig. 4B). The apidaecin was characterized by LC-MS, which showed the purified apidaecin exhibited a molecular weight (2233.75 Da), which conformed to the theoretical value of 2234.68 Da (Fig. 4C). The MS data indicates that the NTG mutagenesis did not change the peptide sequence of apidaecin. Using this method we have calculated an apidaecin heterologous production rate of 418 mg/L after 72 h induction in the pilot-scale fermentation. The MIC was defined as the lowest concentration of the peptide needed for the inhibition of bacterial growth. The MIC of the purified apidaecin toward *E.coli* JM109 was 6.0 mg/L (2.68 uM), similar to previous studies.

## Discussion

Drug resistance is a major problem in antibacterial chemotherapy. Apidaecins can be used as new candidate peptide antibiotics, and are lethal to many Gram-negative bacteria. However, the expensive price of native or chemosynthetic apidaecins is a limiting factor for the clinical application of this AMP. Here we describe for the first time the heterologous expression and secretion of apidaecin by yeast. However, we identified three limiting factors for the high production of apidaecin by yeast. The first of these is the loss of the integration plasmid and loss of cell viability as a result of expression of apidaecin. This suggests that a high concentration of apidaecin is also toxic to yeast. Unlike most conventional AMPs that are amphipathic, apidaecin has a different mode of action to inhibit bacterial growth, and is bacteriostatic rather than displaying membrane-disrupting activity (bacteriolytic action)^27,28^. A mode of action of apidaecin was proposed in which apidaecin binds initially to lipopolysaccharide in the cell membrane and subsequently to the heat shock protein DnaK and related chaperones of *E. coli* in a specific manner^4,29^. *P. pastoris* also expresses a DnaK protein homolog, which shares 59% protein identity with *E.coli*’s DnaK sequence, in the mitochondrial matrix. The toxicity of apidaecin towards *P. pastoris*, which promotes the loss of the integration plasmid and cell viability, may be due to inhibition of this DnaK homolog. We used NTG mutagenesis to obtain a high-yielding apidaecin mutant strain APmu4. It is probable that APmu4 expresses a mutated DnaK homolog protein, which is not sensitive to the high concentration of apidaecin.

A second limiting factor affecting yields is proteolytic degradation of apidaecin, even though the progenitor strain SMD1168 has been optimized for protein expression strain as it lacks protease A activity. Following methanol induction, the present of tryptone and yeast extract is essential to protect against the degradation of apidaecin.

A third limiting factor is the background expression of apidaecin. Previous studies have shown that glucose inhibits the AOX1 promoter activity^25^. As a consequence of apidaecin toxicity to yeast, growing pre-cultures in a medium with glucose is important to reduce the background expression of apidaecin, thereby maximizing growth prior to methanol induction.

**Figure 5:**
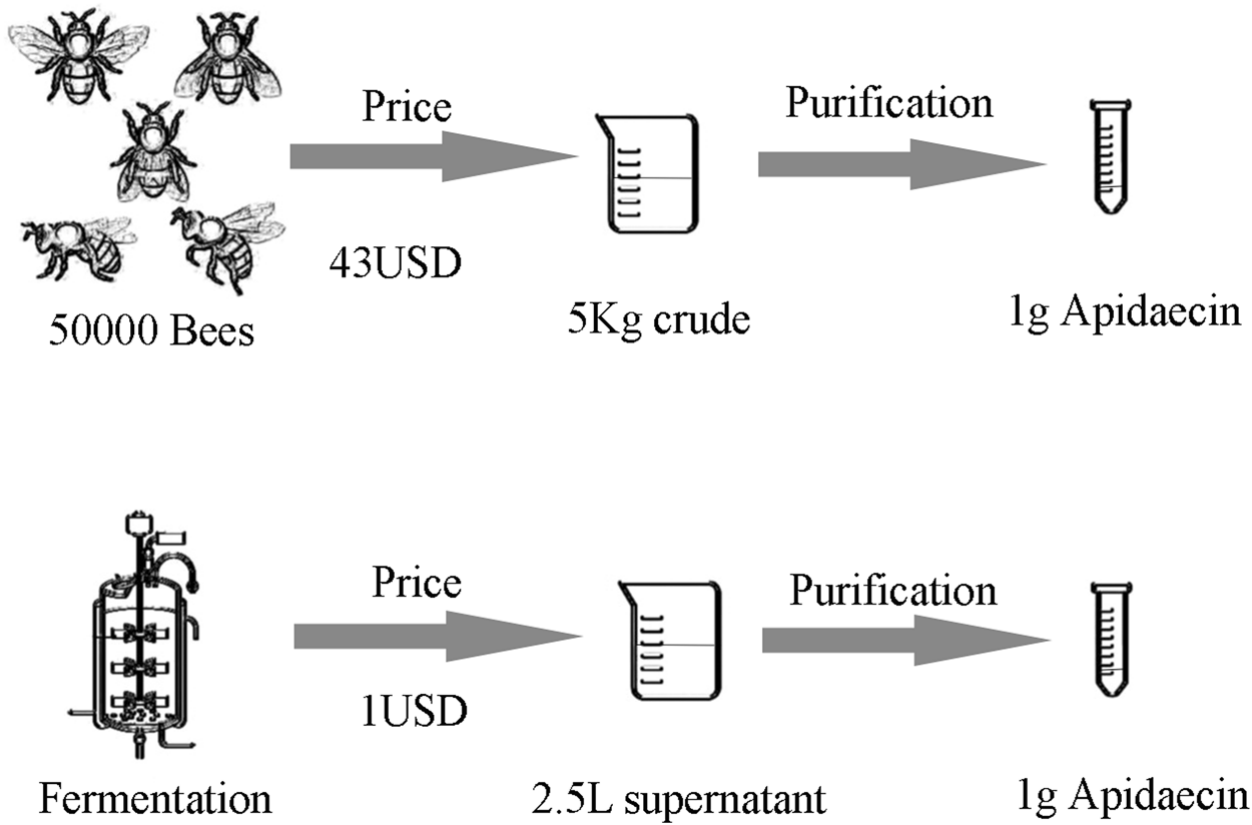
Schematic comparison of the cost for recombinant and extracted natural apidaecin.

We have compared the cost for extracting native apidaecin from bees or obtaining recombinant apidaecin from yeast. As is shown in Fig. 5, a simple and robust pilot-scale fed-batch cultivation process was established and yielded approximately 1 g of apidaecin at only 1USD cost. However, extraction of 1 g of native apidaecin from bees is estimated at 43 USD. Apidaecins have been recognized as potential therapeutic alternatives to antibiotics because of their immediate effect, their apparent nontoxicity toward eukaryotic cells, and the fact that there is little or no bacterial resistance. This study provides a good basis for future economic production of recombinant apidaecin for therapeutic use.

## Methods

### Bacterial strains & plasmid construction

The microorganisms and plasmids used in this work are listed in Table S1. *Escherichia coli* JM109 was used for general cloning purposes and antimicrobial activity tests. All *P. pastoris* strains used in this study are derived from the *P. pastoris* SMD1168, which was used for protein expression^30^. *Escherichia coli* strains were grown aerobically in Luria-Bertoni (LB) broth at 37 °C. Electrocompetent cells of *P. pastoris* were prepared as described^30^. *P. pastoris* strains were cultured in YPD medium, MD medium or BMMY medium (Table S2). During methanol induction, methanol was added daily to attain a 0.5% (vol/vol) final concentration to maintain the induction.

### Construction of pPICAP & strain AP26

Optimized coding sequences for the apidaecin gene was assembled by total gene synthesis according to previously published amino acid sequences. The following primers were used to amplify the apidaecin gene: APP1(AGCTCGAGAAAAGAAGGGTTCGTAGACCGG) and APP2(ATGCGGCCGCTTAAAGTCTAGGATGAGGTGG). The purified PCR product was digested with XhoI and NotI and ligated into pPIC9K digested with the same enzymes. Construction of strain AP26: Strain AP26 was obtained by transferring plasmid pPICAP, which was linearized by SalI, by electroporation. For *E. coli*, ampicillin was used at the 100 ug/ml.

### Detection and quantification of heterologous apidaecin production by antimicrobial assay

The antimicrobial activity of their supernatants was examined by a microtiter plate assay (MPA) and agar diffusion test, using *E.coli* JM109 as the indicator microorganism. Briefly, transformants were first grown on MD plates at 30 °C for 48 h and then transferred into BMMY medium and cultured at 30 °C for different incubation periods. To maintain induction, 100% methanol was added to a final concentration of 0.5% every 24 hours. To test apidaecin production in supernatants of induced cultures, plates of 30 ml of LB agar containing about 10^5^ CFU/ml of the indicator microorganism *E.coli* JM109 were prepared. 50 ul volumes of supernatants of cultures were added into the Oxford cups, and the plates were incubated at 30 °C overnight for the development of inhibition zones. One apidaecin unit (AU) was defined as the reciprocal of the highest dilution of the apidaecin causing 50% growth inhibition (50% of the turbidity of the control culture without apidaecin)^31^. Tricine-sodium dodecyl sulfate polyacrylamide gel electrophoresis (SDS–PAGE) was used to detect apidaecin^32^.

### NTG mutagenesis

AP26 strain was cultured on a MD plate at 30 °C. A single colony was inoculated in a 250 ml flask containing 50 ml of MD liquid medium and incubated at 30 °C until an OD_600_ = 1 was reached. NTG mutagenesis was carried out as described previously with modifications^33^. The cells were collected by centrifugation at 6000 × g for 5 min and washed with 0.05 mol l^−1^ phosphate buffer pH 6.5. The collected cells were resuspended in a buffer solution containing 200 ug ml^−1^ NTG and incubated at 30 °C for 40 min. After the NTG treatment, the cells were washed three times with phosphate buffer and incubated for 6 h in MD liquid medium and then plated onto MD agar and incubated at 30 °C.

### Fed-batch fermentation

The fermentation studies were conducted in10-L bioreactors. A single colony was incubated in MD medium at 30 °C. An overnight culture was inoculated into 200 ml of fresh YDFM medium and cultivated at 30 °C (250 rpm) to an OD_600_ of 4–6. Next, 10% (v/v) inoculum was inoculated into a 10-L fermenter containing 7.0 L BSM medium at 30 °C. The agitation and aeration to operating conditions were the maximum rpm and 1.0 vvm airflow. Glucose was completely consumed after 18–24 h, at which point dissolved oxygen was increased to 100%. With a feed rate of 18.15 ml/h/liter, fed-batch addition of 50% glucose with 1.2% PTM was made for 4 h. At this point additions to final concentrations of 2% and 1%, respectively, of tryptone and yeast extract were made and the pH adjusted to the 6.8 with NH_3_. The methanol induction phase was started by a stepwise increase in the methanol-feeding rate (100% methanol with 12 ml/L PTM1 salts) from 3.6 ml/hr/liter to 10.9 ml/hr/liter over 5–8 h.

### Purification of apidaecin

The culture supernatant was separated, collected by centrifugation at 10,000 × g for 20 min and further sequentially clarified using the 0.22 um, 10 kDa and 1 kDa hollow-fiber membranes. The clarified supernatant was diluted 1:4 with low salt buffer (20 mM sodium phosphate buffer, pH 7.5), and the target proteins were captured using an SP FF column and eluted with 30% buffer B (0.4 M NaCl, 20 mM sodium phosphate buffer, pH 7.5). The eluted proteins were further purified by the HPLC at a flow speed of 1 ml/min. Buffer A used for the HPLC was 0.1% trifluoroacetic in 100% water. Buffer B for the HPLC was 0.1% trifluoroacetic in 100% acetonitrile.

### ESI-MSI

Electrospray ionisation mass spectrometry (ESI-MS) analysis was performed essentially as previously^34,35^.

## Electronic supplementary material

Supplementary Information
