# Supplementary material for: High-level heterologous production and Functional Secretion by recombinant Pichia pastoris of the shortest proline-rich antibacterial honeybee peptide Apidaecin
Source: Sci Rep. 2017 Nov 6;7:14543. doi: 10.1038/s41598-017-15149-3 (PMC5674047; doi:10.1038/s41598-017-15149-3)
Supplement: Supplementary file 1 — Supplementary Information [file 41598_2017_15149_MOESM1_ESM.pdf]

High-level heterologous production and Functional Secretion by recombinant *Pichia pastoris* of the shortest proline-rich antibacterial honeybee peptide Apidaecin

Ximing Chen[ab1], Juan Li [ab1], Haili Sun[c], Shiweng Li[d], Tuo Chen[ab], Guangxiu Liu[ab*], Paul Dyson[e]

[a] Key Laboratory of Desert and Desertification, Northwest Institute of Eco-Environment and Resources, Chinese Academy of Sciences ; China

[b] Key Laboratory of Extreme Environmental Microbial Resources and Engineering of Gansu Province ; China

[c] School of Chemistry and Environment Science, Lanzhou City University; China

[d] School of Chemical and Biological Engineering, Lanzhou Jiaotong University; China

[e] Institute of Life Science, Swansea University Medical School, Singleton Park, Swansea SA2 8PP, UK

* Corresponding authors: To whom correspondence should be addressed.
Email: liugx@lzb.ac.cn

[1] These authors contributed equally to this work.

TABLE S1. Microorganisms and plasmids used in this study

Strain or plasmid	Relevant characteristic	Source or reference
Bacterial strain		
E.coli JM109	Host strain; F *endA1 recA1 gyrA96 thi hsdR17*(r_K^- m_K^+) *relA1 supE44* Δ (lac-*proAB*)<br>F' *traD36 proAB lacIZ* Δ M15	Promega
Yeast (*P. pastoris*) strains		
SMD1168	Host strain; protease A mutant strain for selection on the MD medium; *his4*, *pep4*, Mut$^+$	Invitrogen
C	*P. pastoris* SMD1168 derivative carrying pPIC9K; His$^+$	This work
AP26	*P. pastoris* SMD1168 derivative carrying pPICAP; His$^+$	This work
Plasmids		
pPIC9K	*P. pastoris* 9-kb protein expression and secretion vector carrying a methanol- inducible promoter (PAOX1), 5'AOX1 region, MF α 1_s; His$^+$	Invitrogen
pPICAP	pPIC9K derivative carrying apidaecin fused in frame to MF α 1_s; His$^+$	This work

TABLE S2. Mediums used in this study

**MD**	**BMGY**	**BMGluY**	**YDFM** [34]	**BMM**	**BMMY**	**BSM**
1.34% YNB	1.34% YNB	1.34% YNB	11.83 g/L KH_2PO_4	1.34% YNB	1.34% YNB	26.7 ml/L Phosphoric acid, 85%
4 x 10-5% biotin	4 x 10-5% biotin	4 x 10-5% biotin	2.29 g/L K_2HPO_4	4 x 10-5% biotin	4 x 10-5% biotin	0.93 g/L Calcium sulfate
2% Glucos	1% glycerol	2% Glucose	1 g/L	0.5% methanol	0.5% methanol	18.2 g/L Potassium

e			$MgSO_4$	(ever 24h)	(ever 24h)	sulfate
_	100 mM potassium phosphate , pH 6.0	100 mM potassium phosphate , pH 6.0	10 g/L NH_4SO_4	100 mM potassium phosphate , pH 6.0	100 mM potassium phosphate , pH 6.0	14.9 g/L Magnesium sulfate-7H2 O
_	1% yeast extract	1% yeast extract	0.33mg/ L $CaCl_2$	_	1% yeast extract	4.13 g/L Potassium hydroxide
_	2% tryptone	2% tryptone	1 g NaCl	_	2% tryptone	40.0 g/L Glycerol
_	_	_	1g KCl	_	_	4.35 ml/L PTM
_	_	_	3% Glucose	_	_	_
_	_	_	4.35 ml/L PTM	_	_	_

PTM is the trace salts mix, which contains 6.0 g Cupric sulfate-5H_2O; 0.08 g Sodium iodide; 3.0 g Manganese sulfate-H_2O; 0.2 g Sodium molybdate-2H_2O; 0.02 g Boric Acid; 0.5 g Cobalt chloride; 20.0 g Zinc chloride; 65.0 g Ferrous sulfate-7H_2O; 0.2 g Biotin and 5.0 ml Sulfuric Acid per liter.

TABLE S3. The antimicrobial activity (AU/ml) from supernatants of APmu4 and AP26 incubated with 100ug of chemical synthetic apidaecin in different time

**Incubation time at 30°C**	**AP26 96h**	**AP26 96h+100ug AP**	**APmu4 96h**	**APmu4 96h+100ug AP**
**0h**	32,470±481	1,291,233 ± 35114	154,382±605	1,436,145 ± 45182
**24h**	26,950±325	901,213±9628	122,589±472	976,981 ± 11573

TABLE S4. The production antimicrobial activity (AU/ml) of apidaecin from supernatants of *P. pastoris* APmu4 in different medium

	**BMGY/BMMY**[a]	**BMGluY/BMMY**
**0h**[b]	ND[c]	ND
**24h**	1033±92	1351±118
**48h**	23,519±525	45,382±539
**72h**	341,196±998	735,611±1068

**96h**	164,583 ± 794	549,282 ± 1104

[a] Strain was grown in BMGY or BMGluY and inuction in BMMY at 30°C.

[b] Gene expression was induced at time zero.

[c] ND is no activity detected.

TABLE S5. The production antimicrobial activity (AU/ml) of apidaecin from supernatants of *P. pastoris* APmu4 and AP26

	**APmu4**	**AP26**
**0h[b]**	ND	ND
**24h**	1351 ± 118	1283 ± 108
**48h**	45,382 ± 539	24,589 ± 472
**72h**	735,611 ± 1068	261,910 ± 996
**96h**	549,282 ± 1104	75,331 ± 528

TABLE S6. Pilot-scale fermentation of apidaecin

	**Cell wet weight (g/L)**	**Antimicrobial activity (AU/ml)**
**Batch phase**	92 ± 8	ND[a]
**Glucose feeding phase**	156 ± 13	ND
**Methanol induction 24h**	195 ± 11	3785 ± 211
**Methanol induction 48h**	238 ± 24	852,104 ± 1589
**Methanol induction 72h**	272 ± 18	5,740,361 ± 38520

[a] ND is no activity detected.

TABLE S7. Purification of recombinant apidaecin produced by pilot-scale fermentation

**Purification stage**	**Volume (ml)**	**Total A_{254}**	**Total activity (10^3 AU)**	**Specific activity (AU/ A_{254})**
**Culture supernatant**	100	438.5	5740	13,090
**0.22um hollow-fiber membranes**	100	424	5716	13,481
**10kDa hollow-fiber membranes**	83	28.6	3527	123,322
**1kDa hollow-fiber membrane**	10	220.5	3182	14,431

**Anion exchange chromatography**	3	5.2	2568	493,846
